# Stimulated and unstimulated saliva samples have significantly different bacterial profiles

**DOI:** 10.1371/journal.pone.0198021

**Published:** 2018-06-01

**Authors:** Sonia Gomar-Vercher, Aurea Simón-Soro, José María Montiel-Company, José Manuel Almerich-Silla, Alex Mira

**Affiliations:** 1 University of Valencia, Stomatology Department, Gascó Oliag 1, Valencia, Spain; 2 Department of Genomics and Health, Centre for Advanced Research in Public Health, CSISP-FISABIO, Valencia, Spain; Cairo University, EGYPT

## Abstract

Epidemiological studies use saliva on a regular basis as a non-invasive and easy-to-take sample, which is assumed to be a microbial representative of the oral cavity ecosystem. However, comparative studies between different kinds of saliva samples normally used in microbial studies are scarce. The aim of the current study was to compare oral microbiota composition between two different saliva samples collected simultaneously: non-stimulated saliva with paper points and stimulated saliva collected after chewing paraffin gum. DNA was extracted from saliva samples of ten individuals, then analyzed by 16S rRNA pyrosequencing to describe bacterial diversity. The results demonstrate significant differences between the microbiota of these two kinds of saliva. Stimulated saliva was found to contain an estimated number of species over three times higher than unstimulated saliva. In addition, bacterial composition at the class and genus level was radically different between both types of samples. When compared to other oral niches, both types of saliva showed some similarity to tongue and buccal mucosa, but they do not correlate at all with the bacterial composition described in supra- or sub-gingival dental plaque, questioning their use in etiological and epidemiological studies of oral diseases of microbial origin.

## Introduction

Bacterial communities in the oral cavity contain species that promote health states, while others contribute to disease [1]. Recent studies have shown that poor oral hygiene and/or the presence of specific microorganisms in the oral cavity may be associated with periodontitis, respiratory and intestinal diseases [2, 3, 4]. The kind of samples to be used for such studies, both with epidemiological or etiological purposes, is crucial. Saliva has been the preferred oral sample for decades, as it is considered an easy and non-invasive way to obtain material containing oral bacteria from various locations including mucosal surfaces, supra- and sub-gingival plaque [5, 6, 7]. The salivary microbiota has been used in different human epidemiological studies [8] and has been proposed as a diagnostic marker for oral cancer [9], periodontal disease [10] and dental caries [11]. However, this oral fluid can be collected by different procedures, namely stimulated saliva by chewing sterile paraffin [12, 13, 14], unstimulated saliva by the "spitting method" [15], unstimulated saliva with paper points in floor of the mouth [16, 17] or rinsing with sterile saline [18] among others, but little is known about potential differences among these approaches, and which is the optimal sample kind for each purpose.

Initial culture-based studies proposed that saliva, as it is in contact with all teeth, properly reflects colonization by mutans streptococci in whole dentition [19], but the representativeness of saliva for other caries-associated bacteria which are more fastidious to grow was not known. More recent studies based on PCR-DGGE (Denaturing Gradient Gel Electrophoresis) show a different bacterial profile in saliva and supragingival plaque [20]. When highly parallel tag sequencing methods were used, saliva has been found to be dramatically different from dental plaque in terms of bacterial composition [21]. A recent study performed using Illumina sequencing found that stimulated and unstimulated (drooling) saliva samples from the same individuals were not statistically significant [22]. Another study performed by pyrosequencing showed significant differences in the microbiota of individuals with varying degrees of periodontitis in subgingival plaque samples but not in saliva samples from the same individuals [23], questioning whether salivary samples are representative of the bacterial population at the site where the disease takes place. Similar results were found by Paju *et al*. [24], where no specific bacterial marker for periodontal disease could be established in saliva samples. In a recent work performed by species-specific analysis using the HOMI*NGS* protocol (Human Oral Microbe Identification using Next Generation Sequencing), Belstrøm *et al*. [25] found that stimulated saliva samples provided totally different bacterial profiles compared to site-specific or pooled subgingival samples. However, the levels of the specific periodontal pathogens were detected with comparable accuracy in stimulated saliva samples and in pooled subgingival samples, suggesting that stimulated saliva could be a reasonable alternative in periodontal studies.

High-throughput 16S rRNA sequencing is a more sensitive method than laboratory culture or traditional PCR followed by DGGE or cloning because it provides hundreds or thousands of 16S rRNA reads to describe oral bacterial diversity to an unprecedented level of detail [26]. Although Illumina sequencing provides a large sequencing depth, the longer reads provided by pyrosequencing allow a more accurate taxonomic assignment. The aim of the current study is to determine the oral microbiota composition from stimulated and unstimulated saliva samples from the same individuals by pyrosequencing, and to compare those microbial profiles to the known composition of different oral tissues.

## Materials and methods

### Patient selection and sampling

10 children aged 12 (6 boys and 4 girls, with an average age of 12.7±0.2 years) from a representative sample of the school cohort of the Oral Health Survey of Valencia approved by the Valencian Health Authority in 2010, were randomly selected for saliva sampling. All children received informed consent written and signed by the parents and the study was aproved by Ethics Committee Universitat de València, aproval number H1372162226937.

Intraoral examinations were performed in schools between January and March 2010 and the International Caries Detection and Assessment System (ICDAS) was used as diagnostic criterion for tooth decay. Eight out of the ten had a Decayed, Missing or Filled Teeth Surfaces (DMFS) index of 0 (caries-free, with no history of the disease) and the remaining two a DMFS index >0 (currently caries free, with a history of the disease). The DMFS index average for the 10 children was 1.1 ±1.7. Samples were taken in the morning with an approximate time of bacterial plaque formation of 2–12 hours. Unstimulated saliva samples were collected under the tongue with three sterile paper points ISO 50 deposited for 30 seconds on the mouth floor, and were stored in sterile Eppendorf tubes at -20°C. For stimulated saliva sample collection, individuals were asked to chew paraffin gum for five minutes, then 1 ml of saliva was collected in 20 ml tubes and immediately stored at -20°C.

### DNA amplification and pyrosequencing

DNA extraction was performed from the collected paper points and the stimulated saliva samples using the RTP ® DNA Bacteria Mini Kit (Molecular Stratec, Berlin, Germany), following the manufacturer's instructions. Two PCR amplifications were performed per sample (25 cycles (94°C-10s, 52°C-30s, 68°C-30s) using universal primers 27F and 533R (hypervariable regions V1-V2-V3) containing pyrosequencing adaptors A and B, following Cabrera-Rubio *et al*. 2012. Each sample was amplified using a different forward primer containing a unique identification tag sequence of eight nucleotides, to be used as a "barcode" to distinguish between samples [27]. The PCR products obtained were run on an Agilent bioanalyzer to confirm the absence of nonspecific amplification, and purified by the Ultrapure PCR purification kit (Roche). DNA concentrations were then measured by picogreen fluorescence on a Modulus 9200 fluorimeter (Turner Biosystems) and 20 samples were mixed in equimolar amounts per 1/8^th^ of a plate. Sequencing was performed from the forward end on a Roche GS-FLX pyrosequencing machine (titanium chemistry) at the Centre for Advanced Research in Public Health Research (Valencia, Spain).

### Data analysis

Sequencing reads were separated based on the sample-specific barcodes, end-trimmed and quality-filtered, following Simón-Soro *et al*. 2013. Sequences under 250 bp were also eliminated from the analysis. The sequences were taxonomically assigned using the Ribosomal Database Project (RDP) classifier [28] with an 80% confidence interval, down to the genus level. Assignments to photosynthetic bacteria such as Cyanobacteria were removed, as they are known to correspond to chloroplast DNA from plant-derived food, which is amplified by universal primers [29]. For comparison, 16S rRNA sequences from six oral sites including saliva, available from the Human Microbiome Project database [30] were analyzed by the same procedure. Sequences were clustered at 97% nucleotide identity over 90% sequence alignment length and rarefaction curves were obtained using Mothur [31] with a randomized selection of the same number of sequences per group. Principal Coordinates Analysis (PCoA) was performed with FastUnifrac [32], comparing the 16S-estimated diversity with a phylogenetic approach that takes into account both taxonomically assigned and unassigned reads. Sequences are publicly available at Dryad public data repository with doi:10.5061/dryad.h8c3vq3.

## Results

**Fig 1:**
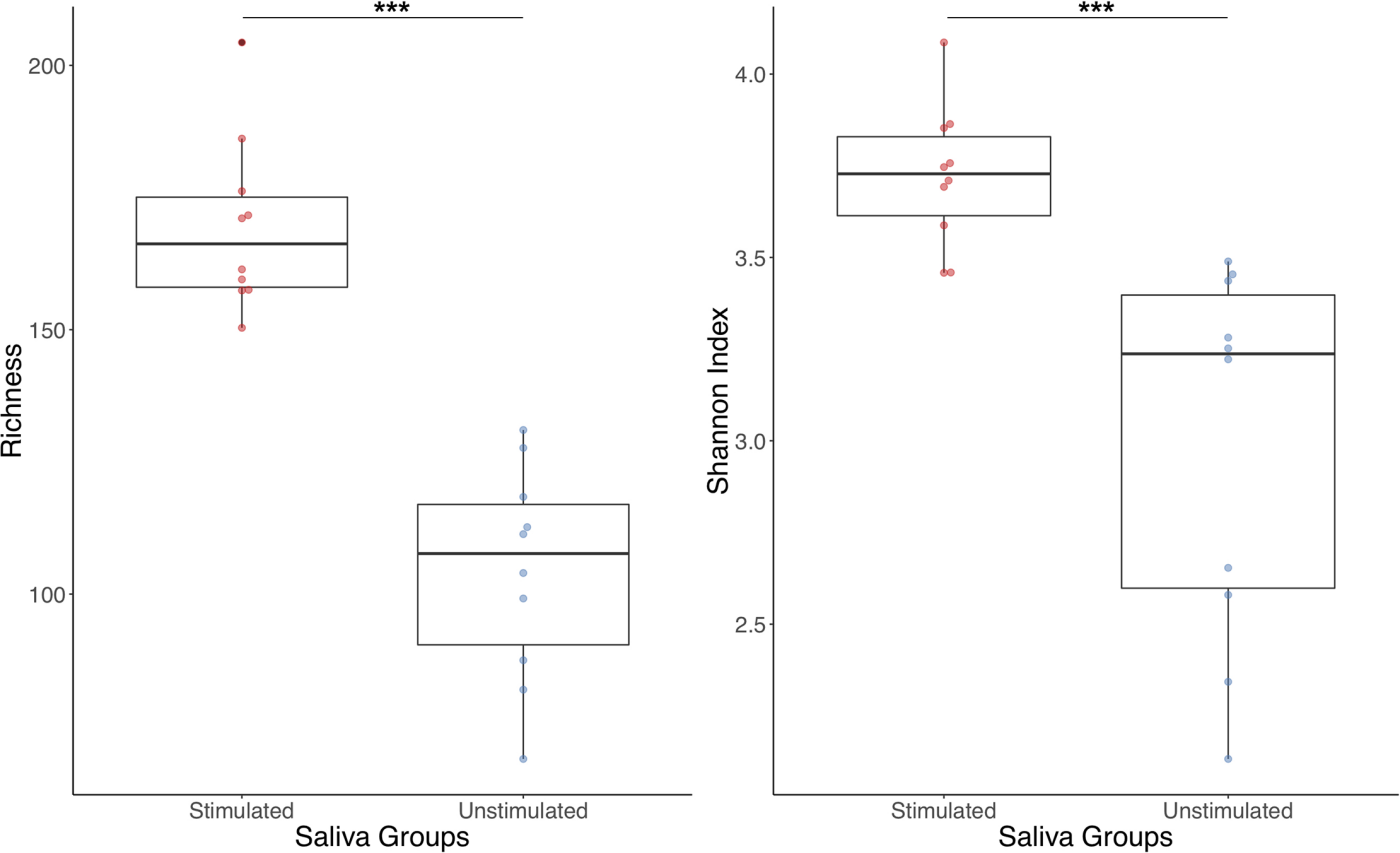
Bacterial alpha-diversity measures in stimulated and unstimulated saliva samples. Boxplots show the estimated number of Operational Taxonomic Units (OTUs) at 97% sequence identity and the Shannon diversity index with the number of sequences rarefied to 1000 reads per sample. Given that a 3% 16S rRNA divergence is the consensus threshold for sequences belonging to the same species (Yarza *et al*., 2008) the estimated richness establish the approximate number of species for a given sequencing effort. Statistically significant differences are marked with asterisks (Wilcox test, p<0.0001 in both cases).

After quality filtering and chimera removal, the average number of reads per sample was 2511. Differences in diversity between stimulated and unstimulated saliva samples were compared with the help of rarefaction curves, which relate the sequencing effort to the estimated number of species, determined by Operational Taxonomic Units (*OTUs*) at 97% of sequence identity, which has been established as the consensus threshold for bacterial species boundaries [33]. The unstimulated saliva curve stabilizes at 600 bacterial species-level *OTUs*, while stimulated saliva shows over 2000 OTUs, indicating that the latter sample type appears to contain a three-fold higher diversity. When richness and diversity indexes were calculated with the number of sequences rarefied to 1000 reads per sample, significant differences were found between the two sample types (Fig 1). Median Shannon indexes were 3,72 for stimulated saliva and 3.23 for unstimulated saliva samples (p = 4.3x10^-5^, Wilcox test). This indicates that in unstimulated saliva samples there are some bacteria that dominate the ecosystem (in this case the *Streptococci*), while in stimulated saliva there is a more even representation of a greater number of oral bacterial species.

**Fig 2:**
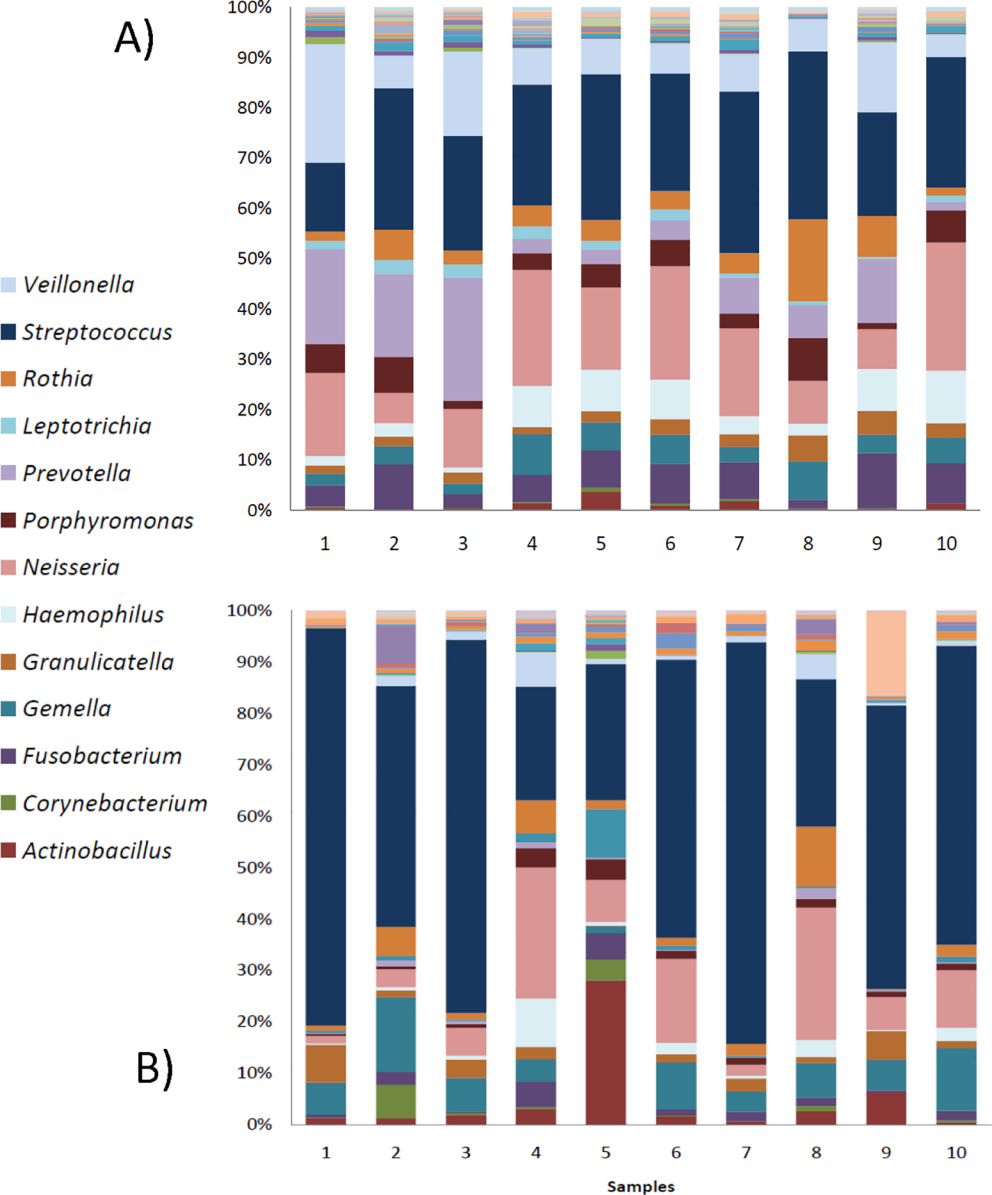
**Salivary bacterial composition at the genus level in stimulated (A) and unstimulated (B) saliva samples.** Markedly different proportions of bacterial genera are observed between the two sample types, including an increased presence of *Streptococcus* in unstimulated saliva, and higher proportions of anaerobic microorganisms in stimulated saliva. Legend indicates those genera present at a proportion >1%.

**Fig 3:**
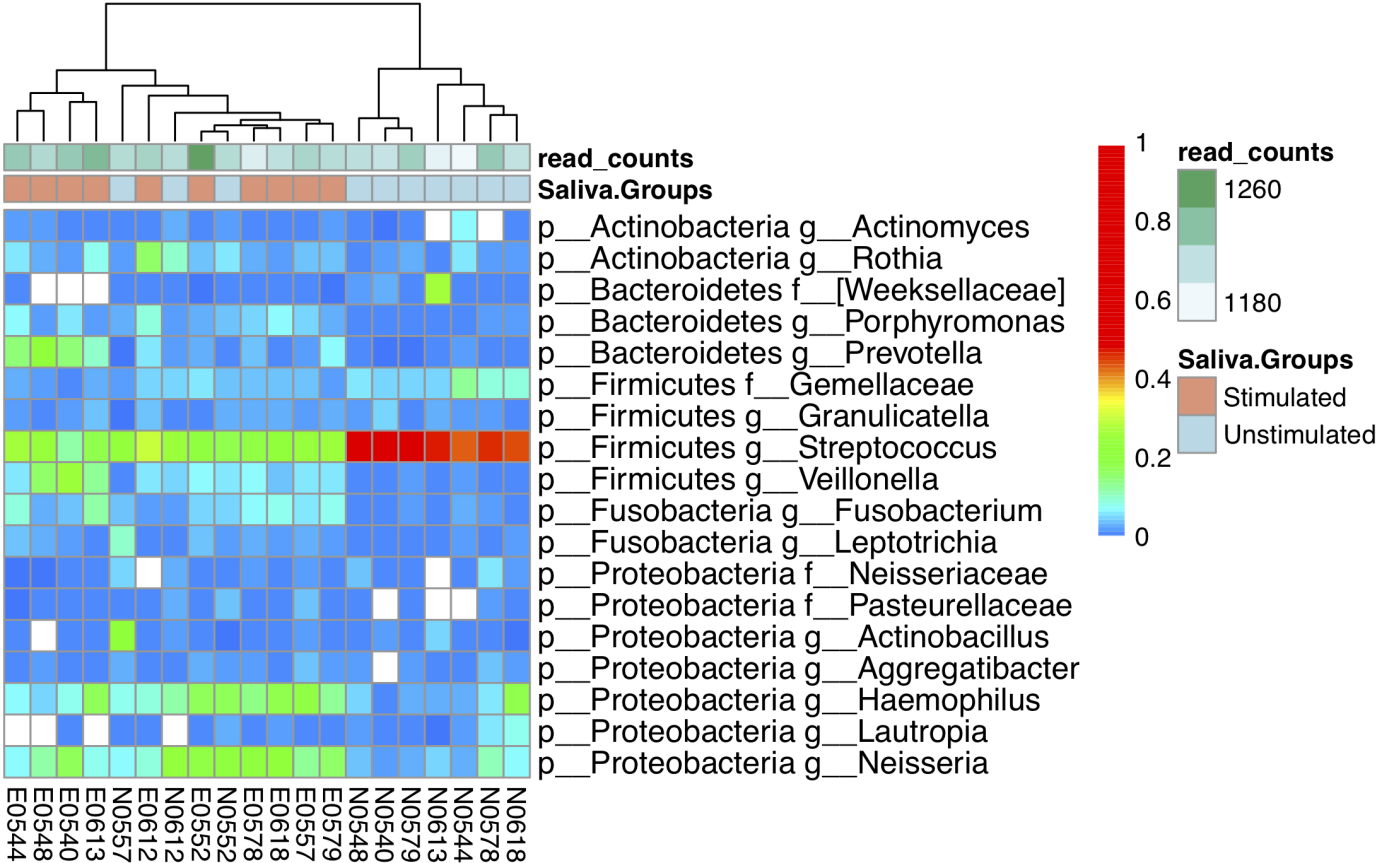
Clustering of stimulated and unstimulated saliva samples according to bacterial composition at the genus level. Heatmap shows the levels of abundant genera in a colour-coded scale. Most samples cluster according to saliva sampling method. Several genera appear over-represented in one or another sample type.

Important differences were observed in the composition of bacterial groups between stimulated and unstimulated saliva samples (Figs 2 and 3), even at high taxonomic ranks. For instance, the proportion of *Bacilli* in stimulated saliva varied from 15 to 40%, while in the unstimulated saliva samples from the same patients they exceeded 50% of the total in 7 of the 10 patients. At the genus level, *Streptococcus* occupies 20–35% of the total sequences in stimulated saliva, followed by *Neisseria* (7–25%), *Prevotella* (2–25%) and *Veillonella* (6–22%) (Fig 2). *Fusobacterium* does not exceed 10% of the total, and the maximum detected level of *Porphyromonas* was 7% of the total. These two typical inhabitants of dental plaque appear in smaller quantities in unstimulated saliva (Fig 2), probably because of plaque removal during chewing of paraffin in stimulated saliva collection. The genus *Streptococcus* is the most abundant in the unstimulated saliva samples, at the expense of many other bacterial genera, which are either at low proportion or absent when compared with stimulated saliva (Fig 3).

**Fig 4:**
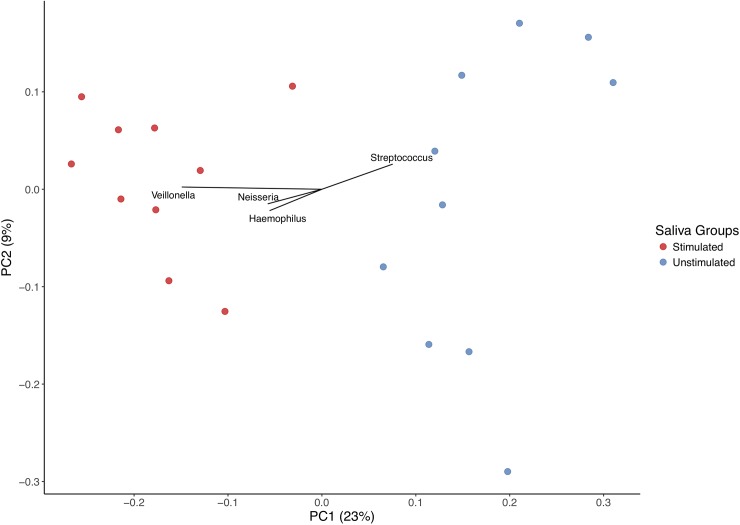
Principal Coordinates Analysis (PCoA) of saliva samples according to bacterial composition. The two principal components of the analysis account for 32% of data variability. Samples do not cluster by donor. However, the principal component clearly separates stimulated from non-stimulated saliva samples. PCoA was performed with UNIFRAC analysis (Lozupone et al. 2006), using the unweighted option with clustering at 97% sequence identity. Vectors showing genera present at >5% proportion are shown, for reference.

The dramatic differences in bacterial composition between the two saliva samples from the same individuals are readily observed by a Principal Coordinates Analysis (*PCoA*), where samples are closer or further from each other in a multidimensional space depending on their degree of similarity in bacterial community structure. When the 20 samples are plotted, the principal component of the *PCoA* clearly separates stimulated and non-stimulated samples from each other, occupying a different position in the 2D space (Fig 4), indicating that microbial composition is unequivocally different (p = 0.001, PERMANOVA test with 1000 permutations). Both weighted and unweighted *PCoA* analyses produced similar results.

**Fig 5:**
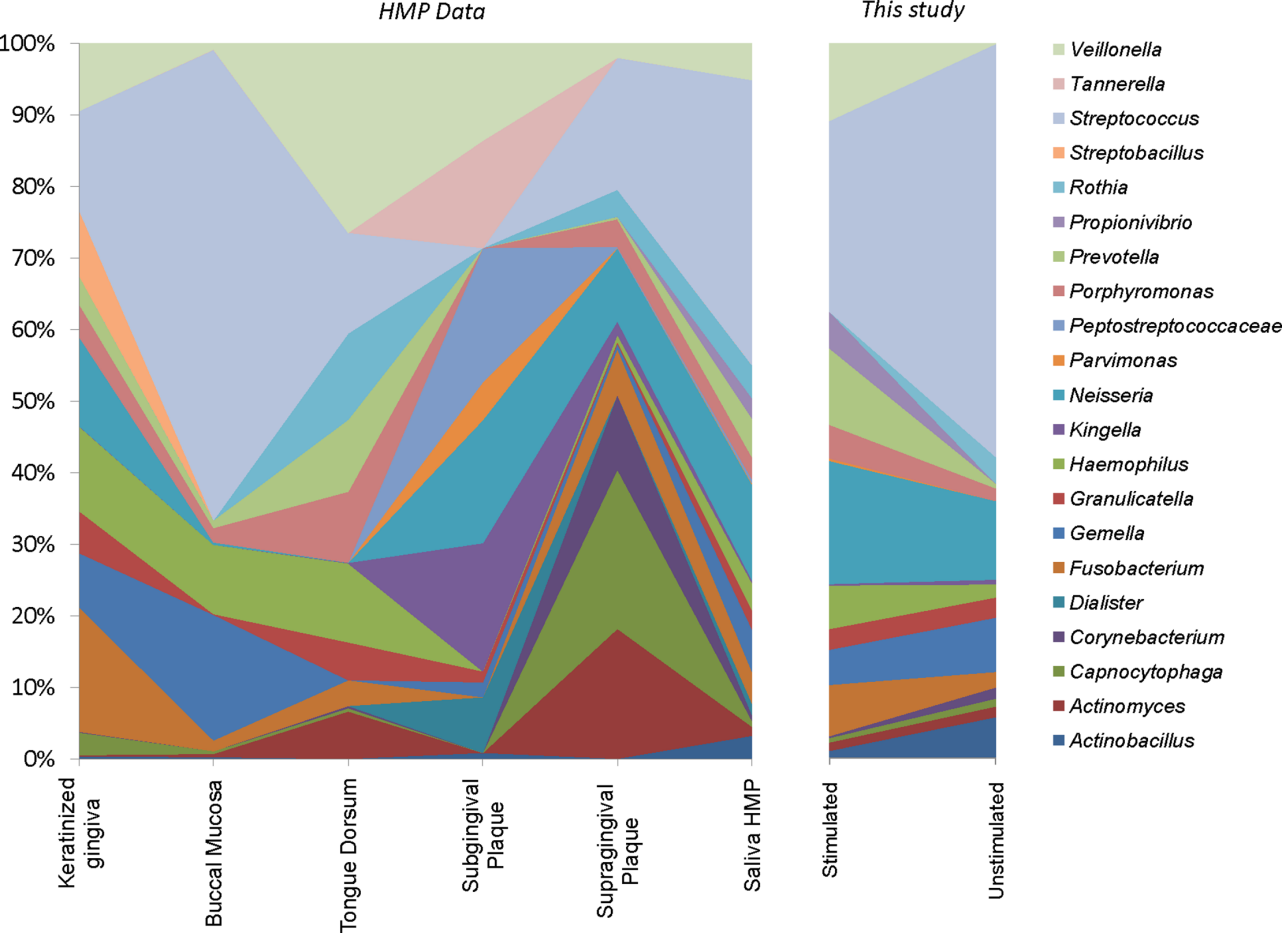
Picassian plot showing bacterial composition. Estimated by pyrosequencing of the 16S rRNA gene, in different oral compartments sampled by the Human Microbiome Project (Segata *et al*. 2012), compared to the proportions in stimulated and unstimulated saliva samples from this study. All samples were analyzed by the same protocol. Saliva from the HMP was collected by a mixture of stimulated and unstimulated procedures (HMP 2012). Data show that saliva samples are not representative of the microbial composition present in subgingival or supragingival dental plaque.

In order to determine whether either saliva sample kind was representative of the microbiota present in different oral niches, their bacterial composition was compared to that found by the Human Microbiome Project (*HMP*) in keratinized gingiva, buccal mucosa, tongue dorsum, subgingival plaque and supragingival dental plaque, as well as saliva [30]. The 16S rRNA pyrosequences obtained by the *HMP* in over 100 individuals comprise the largest set of molecular data in the human oral cavity to date, and therefore represent the best available estimate of bacterial composition in different oral compartments [34]. We extracted the available sequences and analyzed them with the same pipeline used in our saliva samples. The results show that saliva sampled by both collection methods has some similarity with the microbiota composition of buccal mucosa and tongue dorsum, suggesting that those tissues could be one of the main sources of bacteria in saliva (Fig 5). However, from an applied point of view, it is important to note that the microbial composition of saliva is extremely different from that of sub- and supragingival plaque, existing several plaque genera which are absent in saliva and vice versa (Fig 5). Thus, saliva samples are not representative of the microbial profile found at the sites where dental caries, gingivitis and periodontal disease take place.

It is also interesting to note that the saliva samples from the *HMP* [34] were initially collected by a drooling, unstimulated protocol. However, when no sufficient material was obtained, donors were asked to chew paraffin gum to produce stimulated saliva. Thus, the *HMP* saliva samples are a mix of stimulated and unstimulated saliva. This is reflected in the comparison of *HMP* saliva composition to the samples collected in the present study (Fig 5): most bacterial genera in the HMP saliva data show intermediate values between stimulated and unstimulated saliva from our study, highlighting the importance of an appropriate and systematic sampling method to obtain reliable and comparable data.

## Discussion

Many epidemiological studies have routinely taken unstimulated saliva as a representative average of the entire ecosystem of the oral cavity [32, 35]. However, there have been few comparative studies with other kinds of saliva samples [21, 22] and more information is needed to determine whether saliva is an appropriate proxy of microbial composition at the sites of oral diseases.

In this study, we observed profound differences in diversity and taxonomic composition between samples of unstimulated and stimulated saliva from the same individuals. A dramatic 3-fold increase in bacterial diversity was found in stimulated saliva samples (Fig 1). A possible reason of this phenomenon can be the removal of bacterial biofilms attached to different surfaces of the oral cavity, especially the tongue [21] during paraffin gum chewing. The presence of bacteria from subgingival plaque in stimulated saliva samples (Fig 2) suggests that the mechanical forces during chewing may release bacteria from the gingival sulcus, making this collection method more appropriate for detecting periodontal pathogens as proposed by [25]. Nevertheless, many common inhabitants of the subgingival plaque are absent in both saliva samples (Fig 5) and studies that attempt to relate the salivary microbiota to different periodontal health conditions based on (specially unstimulated) saliva may have reduced diagnostic power. Thus, the kind of sample used in epidemiological studies can determine whether significant microbial correlations between health states are found and saliva samples may fail to identify microbial biomarkers of the disease because of its lack of representativeness of bacterial profiles at disease sites.

In dental plaque formation, early colonizers are predominantly Streptococci, and in less amounts, *Neisseria*, *Actinomyces* and *Haemophilus*. When these have colonized the first layers of dental plaque, *Fusobacterium* and *Veillonella* increase in proportion [36]. This could explain the inverse relationship between the proportion of *Streptococcus* and *Fusobacterium-Porphyromonas* observed in our study, which may reflect different stages in plaque formation of each individual (i.e. time since last tooth brushing). A possible antagonistic effect between *Streptococc*i and *Fusobacteria*, due to the sensitivity of the latter to hydrogen peroxide [37] cannot be excluded. Thus, not only saliva collection method but also time of sampling should be standardized for studies from different researchers to be comparable.

Our data show that bacterial proportions in saliva are not correlated to those normally found in dental plaque. This may be the reason why neither tests targeting salivary bacteria nor salivary bacterial tests in combination with clinical parameters have been able to adequately predict the course of caries *in vivo* (for reviews, see [38, 39]). Following the results of our study, we cannot recommend the use of saliva as a representative sample of the oral microbiota at disease sites, especially at specific diseased sites [25]. Nevertheless, it has to be kept in mind that our saliva samples were taking from different individuals to those for which the different oral sites were sampled (age definitely influences bacterial composition), and future studies should collect saliva and plaque samples from the same individuals in order to determine if a given saliva sampling procedure provides appropriate microbial profiles to study oral diseases.

Our results contradict to Belstrøm *et al*. 2016 [22] who state that microbial profiles of unstimulated and stimulated saliva samples collected from the same person are not statistically significantly different. A possible reason for the difference is the use of drooling saliva in their study and paperpoints in ours, as well as the previous thorough flushing with tap water performed by Belstrøm and colleagues in both types of samples. In addition, they discarded the initial stimulated saliva, collecting expectorated saliva for 3 minutes after chewing was finished [22]. In addition, their taxonomic assignment method is based on BLAST on a defined species-level database where important oral species such as *Streptococcus mitis*, *S*. *oralis* or *S*. *dentisani*, among others, are not included, and this could have lowered their diversity estimates. Future research should clarify this point of disagreement, which illustrates the enormous differences in sampling methodology, duration of saliva collection, sampling protocol and sequence analysis pipelines which need to be standardized to provide comparable results.

In studies related to dental caries, saliva was proposed as a more appropriate sample than plaque because the latter was not found reliable in predicting the prevalence of Mutans streptococci due to the variable presence between surfaces [39], but this could be solved by the use of pooled samples from different teeth, at least an incisor, a canine, a premolar and a molar from two opposite quadrants [21].

Past work has failed to consistently show microbial shifts in saliva samples according to caries or periodontal status. The current work suggests that this can be due to the low presence of important inhabitants of plaque in saliva, to the dominant presence of bacteria from oral sites unrelated to caries or periodontitis (for instance bacteria from oral mucosa) and to differences between saliva collection methods that make results inconsistent across studies. In conclusion, the absence of a definitive correlation between salivary bacteria and caries or periodontitis does not mean that a bacterial shift does not take place at the site of the disease, suggesting the use of dental plaque samples in future microbiological studies with diagnostic, etiologic or epidemiological purposes [40].
